# Monument over Hahnemann’s Grave

**Published:** 1898-10

**Authors:** Bushrod W. James

**Affiliations:** N. E. Cor. Eighteenth and Green Streets, Philadelphia, Pa.


### MONUMENT OVER HAHNEMANN'S GRAVE.

N. E. COR. EIGHTEENTH AND GREEN STREETS,
PHILADELPHIA, PA., September 16th, 1898.

TO THE HOMŒOPATHIC PROFESSION AND THE PATRONS OF HOMŒOPATHY IN THE UNITED STATES.

The last International Homœopathic Medical Congress, held in London in 1896, decided to erect a memorial tablet or statue over the remains of the late Dr. Samuel Hahnemann, and accordingly appointed a commission of five to solicit and collect funds for the same.

As the American representative of the Commission, appointed by that Congress, I hereby solicit such voluntary offerings as you desire to contribute toward this object in memory of the illustrious founder of Homœopathy.

The funds are now being contributed, and I would be glad to have all who feel inclined to aid in this matter send in their subscriptions at an early day, either to me or direct to the Secretary, Dr. Francois Cartier, 18 Rue Vignon, Paris, France.

The adornment of the tomb will depend on the amount of cash received, and the Commission desires to proceed at once with the work, in order that it may be finished before the session of the next Homœopathic Medical Congress in Paris in 1900.

Fraternally and sincerely yours,

BUSHROD W. JAMES.

